# GSTM1/GSTT1 double-null genotype increases risk of treatment-resistant schizophrenia: A genetic association study in Brazilian patients

**DOI:** 10.1371/journal.pone.0183812

**Published:** 2017-08-24

**Authors:** Denise S. Pinheiro, Rodrigo da S. Santos, Rodrigo B. de Brito, Aline Helena da S. Cruz, Paulo C. Ghedini, Angela A. S. Reis

**Affiliations:** 1 Department of Biochemistry and Molecular Biology, Institute of Biological Sciences (ICB II), Federal University of Goiás (UFG), Goiânia, GO, Brazil; 2 Department of Nature Sciences (LEdoC), Special Academic Unit of Human Sciences, Federal University of Goiás (UFG), Goiás, GO, Brazil; 3 Brain Institute, Bueno Medical Center, Goiânia, GO, Brazil; 4 Department of Pharmacology, Institute of Biological Sciences (ICB II), Federal University of Goiás (UFG), Goiânia, GO, Brazil; Nagoya Mental Clinic, JAPAN

## Abstract

**Background:**

The role of oxidative stress in schizophrenia has been demonstrated, particularly in subjects with treatment-resistant schizophrenia (TRS). In such patients, the decreased levels of antioxidants in conjunction with the increased generation of reactive oxygen species in the brain exposes the neurons to a higher risk of damage.

**Methods and findings:**

We evaluated the association of deletion polymorphisms of two genes of the antioxidant Glutathione S-Transferase family, *GSTT1* and *GSTM1*, with susceptibility to TRS. A total of 54 TRS patients (mean age 38.7 years) and 78 healthy control subjects (mean age 39.0 years) were enrolled in this study. The subjects were matched by sex, age, and smoking and alcohol consumption habits. In the case group, the frequencies of *GSTT1-*null and *GSTM1*-null genotypes were 24.1 and 51.9%, respectively, whereas for the control group, the frequencies were 12.8 and 46.2%, respectively. Analysis performed with respect to the risk of developing TRS associated with the *GSTT1* and *GSTM1* deletion polymorphisms, resulted in odds ratio (OR) values of 2.1 and 1.2, respectively. However, the association was not found to be significant (p = 0.1229 and p = 0.5916, respectively). The analysis performed with respect to the combined genotypes of *GSTT1* and *GSTM1* revealed that the double-null genotype confers a 4.6-fold increased risk of developing TRS (p = 0.0412).

**Conclusion:**

The results of the present study indicate that a combination of GST deficiencies may play a role in enhanced susceptibility to TRS, and the present genotype of one of these genes may buffer the deficiency caused by the lack (null genotype) of the other. The results suggest that combined deletion polymorphisms of *GSTT1* and *GSTM1* can have implications in the prediction of the clinical course of the disease.

## Introduction

Schizophrenia is a severe disabling mental disorder that affects 0.4 to 0.7% of the general population [1] and is caused by the interaction of multiple genetic and environmental factors. The characteristic symptoms include delusional beliefs, hallucinations, disorganized communication, and deficits in emotional and social behavior. Refractory or treatment-resistant schizophrenia (TRS) is a common condition affecting at least one-third of the patients, in which patients remain symptomatic despite treatment with conventional antipsychotics. Clozapine remains the mainstay of treatment for this condition [2,3].

As the consumption of oxygen by brain cells is very high (approximately 20% of the total body consumption), this can lead to generation of increased levels of reactive oxygen species in the brain, expositing the neurons to oxidative stress [4,5]. This fact, along with the evidence that the antioxidant status is compromised in patients with schizophrenia [6–9], allows us to hypothesize that differences in the ability to deal with oxidative stress can be a risk factor for the development of mental disorders such as schizophrenia.

Increased lipid peroxidation and neuronal damage has been demonstrated in TRS patients, compared to non-refractory cases [10]. It appears that TRS patients are affected to a greater extent by the impairment of the antioxidant system. This prompted us to study this specific group of schizophrenic patients.

The genetic factors have an important role in schizophrenia, as it has been observed that the disease is familial, with a high heritability of about 80% [11]. It is well-known that the genetic nature of schizophrenia is complex, being determined by the interaction of multiple genes and only a small contribution of individual genes [11–13]. In such a scenario, studies regarding polymorphisms that alter the activity of the antioxidant defense system might help in elucidating the mechanisms underlying genetic susceptibility to mental disorders, such as schizophrenia and its refractory subtype.

Glutathione S-transferases (GSTs) are a multigenic superfamily of detoxifying enzymes, involved in the phase II biotransformation of a wide range of compounds, including products generated by oxidative damage and the conjugation of reduced glutathione (GSH) to electrophilic species. Two members of the GST family, *GSTT1* and *GSTM1*, exhibit a well-established deletion polymorphism that leads to a non-functional null allele. In homozygote form, the null allele results in an absence of active isoforms, and the genotypes are called *GSTT1*-null and *GSTM1*-null [14,15].

The null genotype of *GSTT1* and *GSTM1* has been associated with an increased susceptibility to several types of multifactorial conditions in which oxidative stress may be involved, such as diabetes mellitus [16] and many types of cancer, including brain tumors [17]. Recent studies have shown an association between GSTM1/T1 deletion polymorphisms and an increased susceptibility to schizophrenia [18–20]. However, there are considerable inconsistencies in the results and, in particular, the treatment-resistant profile remains an unexplored field with regard to these polymorphisms. Teo et al. (2012) [21] evaluated 384 single nucleotide polymorphisms in candidate genes but did not report significant association with TRS. The study emphasized the necessity of genetic association studies, focused on the treatment-resistant phenotype of schizophrenia, to aid the discovery of specific genetic markers that can help advance the development of individualized treatments.

We evaluated two antioxidant enzymes that are members of the GST family, which are known to have a deletion polymorphism, *GSTM1* and *GSTT1*, in TRS patients from Brazil diagnosed according to American Psychiatric Association criteria [22], to achieve a better understanding of the contribution of these genes to the development of schizophrenia and its refractory manifestation. This study provides evidence for the effect of an important GST deletion polymorphism on TRS.

## Materials and methods

### Subjects

In this study, we enrolled 54 patients (age, 38.67 ± 9.9 years; range, 18 to 65 years) from the Brain Institute, Goiânia, Goiás, Brazil diagnosed with schizophrenia according to the criteria mentioned in the Diagnostic and Statistical Manual of Mental Disorders IV (DSM-IV) published by American Psychiatric Association [22] and considered to have TRS after non-response to two different antipsychotic trials for at least 6 weeks. All patients selected were being treated with clozapine for at least one year to ensure the diagnosis of TRS and to obtain a homogenized group of patients. All of them consented to be a part of the study and neither of them drop out. Clinical data for each patient was extracted from their record file for the period August 2013 to July 2014. A total of 78 healthy individuals (age, 39.03 ± 8.06 years; range, 22 to 56 years) selected from the general population without a history of drug abuse and psychiatric or psychotic disorders including schizophrenia, bipolar disorder, and major depression, were enrolled as a control group matched to the patients by age and sex. From 140 individuals approached, 35 were excluded for having any mental disorder or first- or second-degree relatives with schizophrenia or involvement in substance abuse and 27 for having less than 20-years-old or more than 60-years-old. Data from case and control groups can be seen in S1 File.

This study was approved by the Ethics in Research Committee at the Federal University of Goiás under protocol number 39/2013. All participants answered a health questionnaire and signed an informed consent document after being informed about the research. The questionnaire used for the control group was pretested in a sample of patients and relatives or companions at the reception of the psychiatric clinic before the test in study participants, and was based on the self-report of a previous diagnosis of mental illness. For the case group, the data were extracted from the record file of the patients indicated by the psychiatrist and only personal data and data about consumption of alcohol and tobacco were asked directly to them. The translated and untranslated versions of the questionnaires used can be seen in S2 and S3 Files, respectively. All protocols described here were performed in strict accordance with Ethical Principles for Medical Research Involving Human Subjects of the World Medical Association Declaration of Helsinki.

### Genotyping of *GSTM1* and *GSTT1* polymorphisms

Peripheral blood samples were collected in tubes containing EDTA and stored at -20°C. DNA was extracted using the Illustra Blood Genomic Prep Mini Spin Kit (GE Healthcare^®^, USA). The deletion polymorphism of *GSTM1* and *GSTT1* was determined using a multiplex Real-Time PCR (SYBR Green) followed by melting curve analysis, and confirmed with conventional multiplex PCR and gel electrophoresis, as previously described [16,23].

### Statistical analysis

Statistical analysis was performed using Biostat version 5.3 software. The demographic and clinical characteristics of the participants were compared using a t-test or chi-square test. The genotype frequencies of the two groups were compared using the chi-square test, and Fisher's exact test was applied when necessary. The odds ratios (OR), with their corresponding 95% confidence intervals (CI), and p-values were calculated in order to estimate the risk of developing TRS by multiple logistic regression controlled for the confounding factors sex, smoking, and alcohol consumption. All tests were performed using p < 0.05 as the level of significance.

## Results

A total of 54 TRS patients treated with clozapine (35 male and 19 female) and 78 healthy control subjects (48 male and 30 female) were enrolled in this study. The mean age was 38.7 years for patients and 39.0 years for the control group. Demographic and clinical characteristics of case and control groups are outlined in Table 1.

**Table 1:** Characteristics of the study population and a comparison of case and control groups.

Variables	Case (N = 54)	Control (N = 78)	*P*
Sex (M/F)	35/19	48/30	0.8416
Age (years)	38.67 ± 9.90	39.03 ± 8.06	0.8193
Clozapine dosage (mg/d)	537.04 ± 157.27	--------	--------
Alcohol Consumption (+/-)	6/48	12/66	0.6560
Smoking Habits (+/-)	22/32	23/55	0.2484

Data are reported as mean ± standard deviation. Statistical analysis by t test or chi-square test. Level of significance, p <0.05.

The distribution by sex and age did not show significant differences, indicating homogeneity between the groups. Furthermore, there were no significant differences between the case and control groups in terms of proportion of smokers and drinkers, and there was no association observed with respect to these variables in all the analyses performed. Only those individuals who smoked for at least one year of their life before being diagnosed with schizophrenia in the test group were considered smokers. All subjects reported drinking only occasionally or socially.

The data regarding the *GSTM1* and *GSTT1* genotype frequencies in TRS patients and controls are shown in Table 2. In the test group, the frequencies of *GSTT1-*null and *GSTM1*-null genotypes were 24.1 and 51.9%, respectively, whereas for the control group, the frequencies of *GSTT1-*null and *GSTM1*-null genotype were 12.8 and 46.2%, respectively. There was no difference in the genotypic distribution between case and control groups for both polymorphisms, as confirmed by the chi-square test.

**Table 2:** Distribution of genotypic frequencies of *GSTM1* and *GSTT1* in the study population and a risk analysis performed with respect to TRS.

Genotype	Case n (%)	Control n (%)	Χ²	*P*	OR (IC 95%)	*P*
***GSTM1***						
Present (+)	26 (48.1)	42 (53.8)	-------	-------	1 (Reference)	-------
Null (-)	28 (51.9)	36 (46.2)	0.2180	0.6406	1.22 (0.59–2.51)	0.5916
***GSTT1***						
Present (+)	41 (75.9)	68 (87.2)	-------	-------	1 (Reference)	-------
Null (-)	13(24.1)	10 (12.8)	2.0810	0.1492	2.08 (0.82–5.26)	0.1229
**Total**	54 (100)	78 (100)				

Analysis by chi-square and multiple logistic regression to obtain adjusted odds ratio values (OR) and confidence intervals (95% CI). Level of significance, p < 0.05.

In the multiple logistic regression analysis performed with respect to the risk associated with the *GSTT1* and *GSTM1* deletion polymorphisms of developing TRS, OR values of 2.1 and 1.2 were obtained, respectively. However, the association of these polymorphisms with susceptibility to disease was not significant (p = 0.1229 and p = 0.5916, respectively) in the population studied (S2 Table).

Data regarding the analysis performed with respect to the combined genotypes of *GSTT1* and *GSTM1* are shown in Table 3. A higher percentage of individuals with a double-present genotype (+ / +) was observed for case and control groups (40.7 and 44.9%, respectively). The frequency of individuals who had a double-null genotype (- / -) was low, for both case and control groups (16.7 and 3.8%, respectively). It was possible to verify a significant association for the double-null genotype (- / -), which was more frequently observed in the case group (p = 0.0278). This genotype was associated with a 4.6-fold increased risk for developing TRS (p = 0.0412), compared to the double present genotype (+ / +).

**Table 3:** Distribution frequencies of genotype combinations of *GSTM1* and *GSTT1* in case and control groups and a risk analysis performed with respect to treatment-resistant schizophrenia (TRS).

Genotype GSTT1/GSTM1	Case n (%)	Control n (%)	Χ²	*P*	OR (IC 95%)	*P*
(+ / +)	22 (40.7)	35 (44.9)	------	-------	1 (Reference)	--------
(- / +)	4 (7.4)	7 (9.0)	#	1.000	0.70 (0.16–3.01)	0.6320
(+ / -)	19 (35.2)	33 (42.3)	0.001	0.9812	0.91 (0.41–2.00)	0.8114
(- / -)	9 (16.7)	3 (3.8)	#	0.0278*	4.56 (1.06–19.54)*	0.0412*
Total	54 (100)	78 (100)				

Analysis by chi-square or ^#^Fisher's exact test and multiple logistic regression to obtain adjusted odds ratio values (OR) and confidence intervals (95% CI).

*Significant difference between groups (p < 0.05).

## Discussion

In this genetic association study, *GSTM1* and *GSTT1* deletion polymorphisms were evaluated for their association with susceptibility to TRS by comparing the case and control groups. We demonstrated an association between the double-null genotype (*GSTT1*-null/*GSTM1*-null) and an increase in the risk for developing TRS (S3 Table). This finding was confirmed after controlling for sex, smoking, and alcohol consumption habits (S1 Table). Furthermore, these confounding factors were not significantly associated with the risk for TRS.

It was observed that, despite *GSTT1*-null and *GSTM1*-null genotypes alone having no significant association with TRS susceptibility (S2 Table) (OR = 2.1 with p = 0.1229, and OR = 1.2 with p = 0.5916, respectively), analysis performed with respect to the combination of *GSTT1*-null and *GSTM1*-null genotype revealed significant association for the double-null genotype, conferring a 4.6-fold increased risk of developing the disease (S3 Table) (p = 0.0412). It appears that the present genotype of either *GSTT1* or *GSTM1* confers protection, possibly buffering the functional deficiency caused by lack (null-genotype) of the other gene.

This result is unprecedented and is in accordance with the theory of the complex genetics of schizophrenia, emphasizing the influence of the interaction of several genes [11], and indicates that combination of GST polymorphisms may play a role in predisposition to schizophrenia. This theory is partially in agreement with the conclusions drawn by the study conducted by Gravina et al. (2011), which investigated GST polymorphisms (*GSTT1*, *GSTM1*, *GSTA1* and *GSTP1*) in Italian patients but obtained positive associations only for the *GSTT1*-present/*GSTM1*-null combination.

Other studies, such as the ones by Saadat et al. (2007) and Raffa et al. (2013) also found different results. While a study by Raffa et al. (2013) revealed that individuals with the *GSTT1*-null genotype showed a significantly increased risk for developing schizophrenia in a Tunisian population, a study by Saadat et al. (2007) revealed a significant association between the *GSTT1*-present genotype and an increased risk for developing schizophrenia in an Iranian population. Both studies obtained only small OR values for *GSTT1* (0.6 and 0.42, respectively). A meta-analysis conducted by Kim et al. (2015) [24] concluded that *GSTM1* can be a candidate gene conferring susceptibility to schizophrenia in the East-Asian population. The inconsistent results can be explained by the lack of studies specifically evaluating the association of these polymorphisms with the treatment-resistant profile of schizophrenia. This highlights the necessity of conducting more studies with other populations, as the differences in the ethnical composition and environmental conditions can play an important role in the genetic susceptibility to diseases, particularly multifactorial disorders such as schizophrenia.

As a large amount of auto-oxidizable neurotransmitters such as dopamine, epinephrine, and norepinephrine are present in the brain, the capacity of the cell to detoxify the oxidized metabolites can be of great importance toward prevention of cell damage [5]. O-quinones, the product of oxidation of catecholamines, have been postulated to be associated with the process of neurodegeneration involved in the pathogenesis of schizophrenia and Parkinson’s disease [25–27]. As enzymes of the GSTM class are involved in the detoxification of oxidized metabolites (o-quinones) of catecholamines [26,28], unlike the enzyme encoded by *GSTT1*, which is involved in the metabolism of a wide range of compounds [15], we can hypothesis that a summation of deficiencies in the capacity to eliminate oxidized metabolites can lead to the damage of dopaminergic pathways reported to occur in schizophrenia and its refractory manifestation. Studies involving the association of other genes that influence the antioxidant status by the regulation of the GSH levels, could improve the understanding of the genetic nature of schizophrenia. The mechanisms underlying the results obtained in this study and other studies still need to be investigated further.

It has been observed that antipsychotic drugs can increase oxidative stress by altering the levels of antioxidant enzymes and consequently leading to oxidative injury, particularly lipid peroxidation in the brain [29]. Therefore, it was not well-understood whether the compromised antioxidant system is a cause or a consequence of the pathologic process of schizophrenia [8,30], but the results obtained in this study suggest that it is a cause and not a consequence, because we observed that a possible compromise in the antioxidant capacity (double-null genotype) can cause a 4.6-fold increase in the susceptibility toward TRS.

The frequencies of GSTM1-null and GSTT1-null genotypes in the control group (46.2% and 12.8%, respectively) are close to the frequencies obtained in other case-controlled studies carried out in Brazilian population [31,32] (45.7% and 41.3%, for GSTM1; 19.2% and 18.6% for GSTT1, respectively). Small differences, mainly on GSTT1-null, can be due to the specificities of each study, as target disease and, consequently, different criteria for the control group formation.

With regard to the limitations of this study, we can point to the possibility of the control individuals developing schizophrenia. However, we must consider the fact that only 6% of the sample were below 30 years of age. Other limitation is the relatively small sample size of the case group, which can be attributed to the objective of the study, i.e., to specifically investigate patients with a treatment-resistant profile, the refractory subtype of schizophrenia, in an otherwise homogeneous group of schizophrenic patients treated with clozapine.

## Conclusions

Recently, some studies have investigated the role of oxidative stress in schizophrenia, and most of these studies report that the impairment of the antioxidant system and significant changes in the activities of diverse antioxidant enzymes may play a role in the pathogenesis of the disease [6–8, 10, 20, 29]. Our results corroborate this theory and indicate that genetic association studies can be of great help in the identification of biomarkers to aid in the diagnosis of schizophrenia, or more specifically, its refractory subtype.

The inconsistent results observed in the literature point the necessity of conducting new studies in populations of different ethnic/genetic backgrounds to achieve a better understanding of the contribution of *GSTT1* and *GSTM1* genes to the development of schizophrenia and its refractory manifestation. Thus, more meta-analysis studies should be performed.

The results obtained indicate that *GSTT1* and *GSTM1* are candidate genetic markers for susceptibility to TRS, suggesting that the combination of *GSTT1* and *GSTM1* deletion polymorphisms can have implications in the prediction of the clinical course of the disease. The strong association observed between the *GSTT1*-null/*GSTM1*-null genotype and the refractory manifestation of the disease was not observed in other schizophrenia genetic-association studies, which did not explore the treatment response profile. Nevertheless, we should consider the need for more robust studies, with larger sample size, and enrollment of non-refractory schizophrenic patients, to confirm these preliminary findings in the Brazilian population.

## Supporting information

S1 File Data from case and control groups. (DOCX)

S2 File Questionnaires. Translated questionnaires applied to case and control groups.
(DOCX)

S3 File Untranslated questionnaires. Questionnaires in Portuguese applied to case and control groups.
(DOCX)

S1 Table Characteristics of the study population and a comparison of case and control groups. Data are reported as mean ± standard deviation. Statistical analysis by t test or chi-square. Level of significance (p <0.05).
(DOCX)

S2 Table Distribution of genotypic frequencies for *GSTM1* and *GSTT1* in the study population and a risk analysis of TRS. Analysis by chi-square and multiple logistic regression to obtain adjusted odds ratio values (OR) and confidence intervals (95% CI). Level of significance (p <0.05).
(DOCX)

S3 Table Distribution frequencies of genotype combinations between *GSTM1* and *GSTT1* in case and control groups and a risk analysis of TRS. Analysis by chi-square or Fisher's exact test and multiple logistic regression to obtain adjusted odds ratio values (OR) and confidence intervals (95% CI). *Significant difference between groups (p <0.05).
(DOCX)
