# Supplementary material for: GSTM1/GSTT1 double-null genotype increases risk of treatment-resistant schizophrenia: A genetic association study in Brazilian patients
Source: PLoS One. 2017 Aug 24;12(8):e0183812. doi: 10.1371/journal.pone.0183812 (PMC5570380; doi:10.1371/journal.pone.0183812)
Supplement: S1 File — (DOCX) [file pone.0183812.s001.docx]

**S1 File.** **Data from case and control groups.**

| PATIENT ID | AGE (Years) | GENDER (M=1/F=0) | SMOKING HABIT* | ALCOHOL CONSUMPTION* | GSTT1 (Present=1/Null=0) | GSTM1 (Present=1/Null=0) | CLOZAPINE DOSAGE (mg/day) |
| --- | --- | --- | --- | --- | --- | --- | --- |
| SCHY-TR-001 | 32 | 1 | 0 | 0 | 1 | 1 | 600 |
| SCHY-TR-002 | 34 | 1 | 1 | 1 | 0 | 1 | 600 |
| SCHY-TR-003 | 47 | 0 | 0 | 0 | 1 | 1 | 600 |
| SCHY-TR-004 | 47 | 0 | 0 | 0 | 1 | 1 | 300 |
| SCHY-TR-005 | 22 | 1 | 0 | 0 | 1 | 0 | 600 |
| SCHY-TR-006 | 31 | 1 | 0 | 0 | 1 | 1 | 700 |
| SCHY-TR-007 | 32 | 1 | 0 | 0 | 1 | 1 | 600 |
| SCHY-TR-008 | 40 | 1 | 0 | 0 | 1 | 1 | 200 |
| SCHY-TR-009 | 34 | 0 | 0 | 0 | 0 | 0 | 400 |
| SCHY-TR-010 | 39 | 1 | 0 | 0 | 1 | 1 | 500 |
| SCHY-TR-011 | 49 | 1 | 0 | 0 | 1 | 0 | 150 |
| SCHY-TR-012 | 56 | 0 | 1 | 0 | 1 | 0 | 600 |
| SCHY-TR-013 | 38 | 1 | 0 | 0 | 0 | 0 | 800 |
| SCHY-TR-014 | 42 | 1 | 0 | 0 | 0 | 0 | 400 |
| SCHY-TR-015 | 41 | 1 | 1 | 1 | 1 | 0 | 450 |
| SCHY-TR-016 | 30 | 1 | 1 | 0 | 1 | 0 | 400 |
| SCHY-TR-017 | 32 | 1 | 0 | 1 | 1 | 0 | 500 |
| SCHY-TR-018 | 43 | 0 | 0 | 0 | 1 | 0 | 400 |
| SCHY-TR-019 | 40 | 1 | 0 | 0 | 1 | 0 | 500 |
| SCHY-TR-020 | 43 | 0 | 1 | 0 | 1 | 0 | 600 |
| SCHY-TR-021 | 43 | 0 | 0 | 0 | 1 | 0 | 800 |
| SCHY-TR-022 | 36 | 0 | 0 | 0 | 0 | 0 | 400 |
| SCHY-TR-023 | 28 | 0 | 0 | 0 | 1 | 0 | 600 |
| SCHY-TR-024 | 33 | 1 | 0 | 0 | 0 | 1 | 500 |
| SCHY-TR-025 | 23 | 1 | 0 | 0 | 1 | 1 | 800 |
| SCHY-TR-026 | 29 | 1 | 1 | 0 | 0 | 1 | 100 |
| SCHY-TR-027 | 39 | 1 | 0 | 0 | 1 | 0 | 800 |
| SCHY-TR-028 | 61 | 0 | 0 | 0 | 1 | 0 | 600 |
| SCHY-TR-029 | 34 | 0 | 0 | 0 | 1 | 0 | 500 |
| SCHY-TR-030 | 60 | 1 | 1 | 0 | 0 | 0 | 600 |
| SCHY-TR-031 | 30 | 1 | 1 | 0 | 0 | 0 | 500 |
| SCHY-TR-032 | 43 | 1 | 0 | 0 | 1 | 1 | 600 |
| SCHY-TR-033 | 24 | 1 | 0 | 0 | 1 | 1 | 600 |
| SCHY-TR-034 | 43 | 0 | 1 | 1 | 1 | 1 | 400 |
| SCHY-TR-035 | 39 | 1 | 1 | 0 | 0 | 0 | 600 |
| SCHY-TR-036 | 19 | 1 | 0 | 0 | 1 | 1 | 800 |
| SCHY-TR-037 | 36 | 1 | 1 | 0 | 1 | 1 | 600 |
| SCHY-TR-038 | 28 | 0 | 1 | 0 | 1 | 1 | 600 |
| SCHY-TR-039 | 40 | 1 | 1 | 0 | 1 | 0 | 400 |
| SCHY-TR-040 | 40 | 0 | 1 | 0 | 1 | 0 | 800 |
| SCHY-TR-041 | 37 | 0 | 0 | 0 | 1 | 0 | 600 |
| SCHY-TR-042 | 43 | 1 | 1 | 0 | 1 | 1 | 300 |
| SCHY-TR-043 | 40 | 0 | 0 | 1 | 1 | 1 | 600 |
| SCHY-TR-044 | 44 | 0 | 0 | 0 | 0 | 0 | 600 |
| SCHY-TR-045 | 53 | 1 | 0 | 0 | 1 | 0 | 600 |
| SCHY-TR-046 | 35 | 1 | 0 | 0 | 1 | 1 | 600 |
| SCHY-TR-047 | 65 | 0 | 0 | 0 | 0 | 0 | 600 |
| SCHY-TR-048 | 44 | 0 | 1 | 0 | 1 | 1 | 600 |
| SCHY-TR-049 | 26 | 1 | 1 | 0 | 1 | 0 | 400 |
| SCHY-TR-050 | 30 | 1 | 1 | 0 | 1 | 1 | 400 |
| SCHY-TR-051 | 39 | 1 | 1 | 0 | 1 | 1 | 600 |
| SCHY-TR-052 | 31 | 1 | 1 | 1 | 0 | 1 | 600 |
| SCHY-TR-053 | 58 | 1 | 1 | 0 | 1 | 1 | 600 |
| SCHY-TR-054 | 43 | 1 | 1 | 0 | 1 | 1 | 400 |

*Alcohol Consumption - indicate the habit of drinking alcohol at least occasionally for at least one year before the diagnosis of schyzophrenia.

*Smoking Habit - indicate the habit of smoking for at least one year before the diagnosis of schyzophrenia.

| Control ID | AGE (Years) | GENDER (M=1/F=0) | SMOKING HABIT* | ALCOHOL CONSUMPTION* | GSTT1 Genotype (Present=1/Null=0) | GSTM1 Genotype (Present=1/Null=0) |
| --- | --- | --- | --- | --- | --- | --- |
| CON001 | 54 | 0 | 0 | 0 | 1 | 1 |
| CON003 | 52 | 0 | 1 | 0 | 1 | 1 |
| CON006 | 29 | 1 | 0 | 1 | 1 | 1 |
| CON007 | 39 | 1 | 0 | 1 | 0 | 1 |
| CON010 | 47 | 1 | 0 | 0 | 1 | 1 |
| CON013 | 33 | 0 | 0 | 0 | 1 | 1 |
| CON016 | 31 | 0 | 0 | 1 | 1 | 0 |
| CON022 | 39 | 1 | 0 | 1 | 1 | 0 |
| CON024 | 32 | 0 | 1 | 0 | 1 | 0 |
| CON028 | 35 | 0 | 0 | 0 | 1 | 0 |
| CON031 | 56 | 1 | 1 | 1 | 1 | 0 |
| CON033 | 48 | 1 | 0 | 0 | 1 | 1 |
| CON034 | 38 | 0 | 0 | 0 | 1 | 0 |
| CON039 | 35 | 1 | 0 | 0 | 1 | 0 |
| CON040 | 48 | 0 | 0 | 0 | 1 | 0 |
| CON042 | 34 | 1 | 0 | 0 | 1 | 1 |
| CON049 | 42 | 1 | 0 | 0 | 1 | 1 |
| CON051 | 48 | 1 | 1 | 1 | 1 | 1 |
| CON052 | 35 | 0 | 0 | 0 | 1 | 1 |
| CON053 | 24 | 1 | 0 | 0 | 0 | 1 |
| CON054 | 35 | 0 | 0 | 0 | 1 | 0 |
| CON055 | 32 | 1 | 0 | 0 | 1 | 0 |
| CON056 | 29 | 1 | 0 | 0 | 1 | 0 |
| CON057 | 35 | 1 | 0 | 0 | 1 | 1 |
| CON058 | 28 | 0 | 0 | 0 | 1 | 0 |
| CON059 | 44 | 1 | 0 | 0 | 1 | 0 |
| CON060 | 30 | 0 | 0 | 0 | 1 | 1 |
| CON061 | 32 | 1 | 1 | 0 | 1 | 1 |
| CON062 | 45 | 0 | 0 | 0 | 0 | 1 |
| CON063 | 49 | 1 | 1 | 0 | 1 | 0 |
| CON064 | 42 | 1 | 0 | 0 | 1 | 1 |
| CON066 | 32 | 1 | 0 | 0 | 1 | 1 |
| CON067 | 42 | 1 | 0 | 0 | 1 | 1 |
| CON068 | 45 | 0 | 1 | 0 | 1 | 1 |
| CON070 | 28 | 1 | 0 | 0 | 1 | 1 |
| CON071 | 42 | 1 | 0 | 0 | 1 | 0 |
| CON072 | 22 | 1 | 0 | 0 | 1 | 1 |
| CON074 | 37 | 0 | 0 | 0 | 1 | 1 |
| CON075 | 45 | 0 | 0 | 0 | 1 | 1 |
| CON076 | 51 | 0 | 0 | 0 | 1 | 0 |
| CON078 | 42 | 1 | 0 | 1 | 1 | 1 |
| CON079 | 36 | 1 | 0 | 0 | 0 | 0 |
| CON081 | 24 | 1 | 0 | 0 | 0 | 1 |
| CON082 | 40 | 0 | 1 | 0 | 1 | 0 |
| CON085 | 48 | 1 | 0 | 0 | 0 | 0 |
| CON086 | 50 | 0 | 0 | 0 | 1 | 0 |
| CON087 | 48 | 1 | 1 | 0 | 1 | 1 |
| CON088 | 43 | 0 | 0 | 0 | 1 | 1 |
| CON089 | 42 | 0 | 0 | 0 | 1 | 1 |
| CON091 | 37 | 1 | 1 | 1 | 1 | 1 |
| CON092 | 48 | 0 | 1 | 0 | 1 | 1 |
| CON093 | 55 | 1 | 0 | 0 | 1 | 0 |
| CON095 | 31 | 1 | 0 | 0 | 1 | 1 |
| CON096 | 35 | 1 | 0 | 0 | 1 | 1 |
| CON097 | 43 | 1 | 0 | 0 | 1 | 0 |
| CON098 | 50 | 1 | 1 | 0 | 0 | 1 |
| CON099 | 41 | 1 | 1 | 0 | 1 | 0 |
| CON100 | 42 | 1 | 0 | 0 | 1 | 0 |
| CON0101 | 44 | 0 | 0 | 0 | 1 | 0 |
| CON0103 | 49 | 1 | 1 | 0 | 1 | 0 |
| CON0104 | 38 | 1 | 1 | 0 | 1 | 1 |
| CON0106 | 39 | 0 | 0 | 1 | 1 | 1 |
| CON0109 | 46 | 1 | 0 | 0 | 1 | 0 |
| CON0111 | 34 | 1 | 0 | 0 | 1 | 0 |
| CON0112 | 41 | 1 | 0 | 0 | 1 | 1 |
| CON0113 | 28 | 0 | 0 | 1 | 1 | 0 |
| CON0114 | 44 | 1 | 1 | 0 | 1 | 0 |
| CON0115 | 34 | 0 | 0 | 0 | 1 | 1 |
| CON0116 | 30 | 0 | 1 | 0 | 1 | 0 |
| CON0117 | 38 | 1 | 1 | 0 | 0 | 1 |
| CON0118 | 32 | 0 | 0 | 0 | 1 | 1 |
| CON0121 | 43 | 1 | 1 | 0 | 1 | 0 |
| CON0126 | 51 | 0 | 1 | 0 | 0 | 0 |
| CON0130 | 31 | 1 | 0 | 0 | 1 | 0 |
| CON0132 | 31 | 0 | 0 | 0 | 1 | 0 |
| CON0137 | 26 | 1 | 1 | 0 | 1 | 0 |
| CON0138 | 34 | 1 | 1 | 1 | 0 | 1 |
| CON0140 | 32 | 1 | 1 | 1 | 1 | 1 |

*Alcohol Consumption - indicate the habit of drinking alcohol at least occasionally for at least one year during the life.

*Smoking Habit - indicate the habit of smoking for at least one year during the life.
