# Supplementary material for: GSTM1/GSTT1 double-null genotype increases risk of treatment-resistant schizophrenia: A genetic association study in Brazilian patients
Source: PLoS One. 2017 Aug 24;12(8):e0183812. doi: 10.1371/journal.pone.0183812 (PMC5570380; doi:10.1371/journal.pone.0183812)
Supplement: S2 File — Translated questionnaires applied to case and control groups. (DOCX) [file pone.0183812.s002.docx]

**S2 File. Questionnaires.** Translated questionnaires applied to case and control groups.

**QUESTIONNAIRE - Patients Treatment-Resistant Schizophrenia**

INITIALS: ________________ TIME: ________________ ID: SCHY_________

**Personal Data:**

Name: _______________________________________________________________

Date of birth:____/____/____ Age: _____ Sex:______________

Identity document:_____________________ General Registry Entity:___________

Person's certificate: ________________ Marital status:_____________________

Address: _______________________________________________________

Townhouse: _____________________ City: _____________ State: _______

Zip Code: ______________ Fone number: ______________ Cell Phone: ________

Place of Birth: _______________________ Nationality: _______________

- Were you a smoker before the diagnosis of schizophrenia? ( )Yes ( )No

If Yes, for how long? _________

If Yes, how often? ( )Daily ( ) Occasionally

Did you use alcohol prior to the diagnosis of schizophrenia?

( ) Yes ( ) No

If Yes, for how long? _________

If Yes, how often? ( )Daily ( )Socially ( ) Occasionally

**Clinical Data (File Record): Clozapine (mg/day):** ______________

Collection Location:__________________________

Name of the person responsible for completing the questionnaire and signature:

**QUESTIONNAIRE – Control Group**

INITIALS: ________________ TIME: ________________ ID: CON_________

**Personal Data:**

Name: _______________________________________________________________

Date of birth:____/____/____ Age: _____ Sex:______________

Identity document:_____________________ General Registry Entity:___________

Person's certificate: ________________ Marital status:_____________________

Address: _______________________________________________________

Townhouse: _____________________ City: _____________ State: _______

Zip Code: ______________ Fone number: ______________ Cell Phone: ________

Place of Birth: _______________________ Nationality: _______________

- Have you ever had treatment with a psychologist or psychiatrist? ( )Yes ( )No

If Yes, for what reason? ____________________________________________

_________________________________________________________________

- Do you use any medications? ( )Yes ( )No

If Yes, What?_____________________________________________________

_________________________________________________________________

- Have you had any mental problems such as schizophrenia, depression, bipolar disorder and so on? ( )Yes ( )No

If Yes, What? __________________________________________________

_________________________________________________________________

- Are you a smoker or have you ever smoked? ( )Yes ( )No

Se Yes, for how long? _________

Se Yes, how often? ( )Daily ( )Socially ( ) Occasionally

- Have you ever consumed alcohol?

( ) Yes ( ) No

Se Yes, for how long? _________

Se Yes, how often? ( )Daily ( )Socially ( ) Occasionally

- Have you ever used drugs such as marijuana, cocaine, crack, and so on?

( ) Yes ( ) No

Collection Location:__________________________

Name of the person responsible for completing the questionnaire and signature:
