# Supplementary material for: GSTM1/GSTT1 double-null genotype increases risk of treatment-resistant schizophrenia: A genetic association study in Brazilian patients
Source: PLoS One. 2017 Aug 24;12(8):e0183812. doi: 10.1371/journal.pone.0183812 (PMC5570380; doi:10.1371/journal.pone.0183812)
Supplement: S3 File — Questionnaires in Portuguese applied to case and control groups. (DOCX) [file pone.0183812.s003.docx]

**S3 File. Untranslated Questionnaires.** Questionnaires in Portuguese applied to case and control groups.

**QUESTIONÁRIO – Pacientes Esquizofrenia Resistente ao Tratamento**

INICIAIS: ________________ Data ________________ ID: SCHY_________

**DADOS PESSOAIS:**

Nome: _______________________________________________________________

Data de nascimento:____/____/____ Idade: _____ Sexo:______________

Documento de Identidade:_____________________ Org. Exp. ___________

CPF: ________________ Estado Civil: ______________________

Endereço: _______________________________________________________

Bairro: _____________________ Cidade: _____________ Estado: _______

CEP: _______________ Telefone: _______________ Celular: ____________

Naturalidade: _______________________ Nacionalidade: _______________

- Você era fumante antes do diagnóstico de esquizofrenia? ( )Sim ( )Não

Se sim, por quanto tempo? _________

Se sim, qual a frequência? ( )Diariamente ( ) Ocasionalmente

- Você consumia bebida alcoólica antes do diagnóstico de esquizofrenia?

( ) Sim ( ) Não

Se sim, por quanto tempo? _________

Se sim, qual a frequência? ( )Diariamente ( )Socialmente ( ) Ocasionalmente

**DADOS CLÍNICOS (ficha do paciente): Clozapina (mg/dia):** ______________

Local de Coleta:__________________________

Nome do Responsável pelo preenchimento do questionário e assinatura:

**QUESTIONÁRIO – Grupo Controle**

INICIAIS: ________________ Data ________________ ID: CON_________

**DADOS PESSOAIS:**

Nome: _______________________________________________________________

Data de nascimento:____/____/____ Idade: _____ Sexo:______________

Documento de Identidade:_____________________ Org. Exp. ___________

CPF: ________________ Estado Civil: ______________________

Endereço: _______________________________________________________

Bairro: _____________________ Cidade: _____________ Estado: _______

CEP: _______________ Telefone: _______________ Celular: ____________

Naturalidade: _______________________ Nacionalidade: _______________

- Você faz ou já fez tratamento com psicólogo ou psiquiatra? ( )Sim ( )Não

Se sim, por qual motivo? ____________________________________________

_________________________________________________________________

- Você utiliza algum medicamento? ( )Sim ( )Não

Se sim, Qual?_____________________________________________________

_________________________________________________________________

- Você tem ou já teve algum problema mental, como esquizofrenia, depressão, distúrbio bipolar etc? ( )Sim ( )Não

Se sim, Qual? __________________________________________________

_________________________________________________________________

- Você é fumante ou já fumou alguma vez na vida? ( )Sim ( )Não

Se sim, por quanto tempo? _________

Se sim, qual a frequência? ( )Diariamente ( ) Ocasionalmente

- Você consume ou consumia bebida alcoólica?

( ) Sim ( ) Não

Se sim, por quanto tempo? _________

Se sim, qual a frequência? ( )Diariamente ( )Socialmente ( ) Ocasionalmente

- Você já usou drogas como maconha, cocaína, crack etc?

( ) Sim ( ) Não

Local de Coleta:__________________________

Nome do Responsável pelo preenchimento do questionário e assinatura:
