# Supplementary material for: GSTM1/GSTT1 double-null genotype increases risk of treatment-resistant schizophrenia: A genetic association study in Brazilian patients
Source: PLoS One. 2017 Aug 24;12(8):e0183812. doi: 10.1371/journal.pone.0183812 (PMC5570380; doi:10.1371/journal.pone.0183812)
Supplement: S1 Table — Data are reported as mean ± standard deviation. Statistical analysis by t test or chi-square. Level of significance (p <0.05). (DOCX) [file pone.0183812.s004.docx]

S1 Table. Characteristics of the study population and a comparison of case and control groups.

| **Variables** | **Case (N=54)** | **Control (N=78)** | ***P*** |
| --- | --- | --- | --- |
| Sex (M/F) | 35/19 | 48/30 | 0.8416 |
| Age (years) | 38.67 ± 9.90 | 39.03 ± 8.06 | 0.8193 |
| Clozapine dosage (mg/d) | 537.04 ± 157.27 | -------- | -------- |
| Alcohol Consumption (+/-) | 6/48 | 12/66 | 0.6560 |
| Smoking Habits (+/-) | 22/32 | 32/46 | 0.8829 |

Data are reported as mean ± standard deviation. Statistical analysis by t test or chi-square. Level of significance (p <0.05).
