# Supplementary material for: GSTM1/GSTT1 double-null genotype increases risk of treatment-resistant schizophrenia: A genetic association study in Brazilian patients
Source: PLoS One. 2017 Aug 24;12(8):e0183812. doi: 10.1371/journal.pone.0183812 (PMC5570380; doi:10.1371/journal.pone.0183812)
Supplement: S2 Table — Analysis by chi-square and multiple logistic regression to obtain adjusted odds ratio values (OR) and confidence intervals (95% CI). Level of significance (p <0.05). (DOCX) [file pone.0183812.s005.docx]

S2 Table. Distribution of genotypic frequencies of *GSTM1* and *GSTT1* in the study population and a risk analysis performed with respect to TRS.

| **Genotype** | **Case**  **n (%)** | **Control**  **n (%)** | **Χ²** | ***P*** | **OR (IC 95%)** | ***P*** |
| --- | --- | --- | --- | --- | --- | --- |
| ***GSTM1*** |  |  |  |  |  |  |
| Present (+) | 26 (48.1) | 42 (53.8) | ------- | ------- | 1 (Reference) | ------- |
| Null (-) | 28 (51.9) | 36 (46.2) | 0.2180 | 0.6406 | 1.22 (0.59-2.51) | 0.5916 |
| ***GSTT1*** |  |  |  |  |  |  |
| Present (+) | 41 (75.9) | 68 (87.2) | ------- | ------- | 1 (Reference) | ------- |
| Null (-) | 13(24.1) | 10 (12.8) | 2.0810 | 0.1492 | 2.08 (0.82-5.26) | 0.1229 |
| **Total** | 54 (100) | 78 (100) |  |  |  |  |

Analysis by chi-square and multiple logistic regression to obtain adjusted odds ratio values (OR) and confidence intervals (95% CI). Level of significance (p <0.05).
