# Supplementary material for: GSTM1/GSTT1 double-null genotype increases risk of treatment-resistant schizophrenia: A genetic association study in Brazilian patients
Source: PLoS One. 2017 Aug 24;12(8):e0183812. doi: 10.1371/journal.pone.0183812 (PMC5570380; doi:10.1371/journal.pone.0183812)
Supplement: S3 Table — Analysis by chi-square or Fisher's exact test and multiple logistic regression to obtain adjusted odds ratio values (OR) and confidence intervals (95% CI). *Significant difference between groups (p <0.05). (DOCX) [file pone.0183812.s006.docx]

**S3 Table.** **Distribution frequencies of genotype combinations of *GSTM1* and *GSTT1* in case and control groups and a risk analysis performed with respect to treatment-resistant schizophrenia (TRS).**

| **Genotype**  **GSTT1/GSTM1** | **Case**  **n (%)** | **Control**  **n (%)** | **Χ²** | ***P*** | **OR (IC 95%)** | ***P*** |
| --- | --- | --- | --- | --- | --- | --- |
| (+ / +) | 22 (40.7) | 35 (44.9) | ------ | ------- | 1 (Reference) | -------- |
| (- / +) | 4 (7.4) | 7 (9.0) | # | 1.000 | 0.70 (0.16-3.01) | 0.6320 |
| (+ / -) | 19 (35.2) | 33 (42.3) | 0.001 | 0.9812 | 0.91 (0.41-2.00) | 0.8114 |
| (- / -) | 9 (16.7) | 3 (3.8) | # | 0.0278* | 4.56 (1.06-19.54)* | 0.0412* |
| Total | 54 (100) | 78 (100) |  |  |  |  |

Analysis by chi-square or ^#^Fisher's exact test and multiple logistic regression to obtain adjusted odds ratio values (OR) and confidence intervals (95% CI). *Significant difference between groups (p <0.05).
